# Very Low Prevalence and Incidence of Atrial Fibrillation among Bolivian Forager-Farmers

**DOI:** 10.5334/aogh.3252

**Published:** 2021-02-16

**Authors:** Christopher J. Rowan, Michael A. Eskander, Edmond Seabright, Daniel Eid Rodriguez, Edhitt Cortez Linares, Raul Quispe Gutierrez, Juan Copajira Adrian, Daniel Cummings, Bret Beheim, Kirsten Tolstrup, Abinash Achrekar, Thomas Kraft, David E. Michalik, Michael I. Miyamoto, Adel H. Allam, L. Samuel Wann, Jagat Narula, Benjamin C. Trumble, Jonathan Stieglitz, Randall C. Thompson, Gregory S. Thomas, Hillard S. Kaplan, Michael D. Gurven

**Affiliations:** 1 Renown Institute for Heart and Vascular Health, Reno, Nevada, USA; 2 Western Washington Medical Group, Everett, Washington, USA; 3 University of New Mexico, Department of Anthropology, New Mexico, USA; 4 Universidad de San Simon, Bolivia; 5 Tsimane Health and Life History Project, San Borja, Beni, Bolivia; 6 Max Plank Institute of Evolutionary Anthropology, Department of Human Behavior, Culture and Ecology, Leipzig, Germany; 7 Cardiology, Dept. of Medicine, University of California, San Francisco, CA, USA; 8 University of New Mexico, Division of Cardiology, Albuquerque, New Mexico, USA; 9 University of California, Santa Barbara, Department of Anthropology, USA; 10 Miller Children’s and Women’s Hospital Long Beach, CA, USA; 11 Division of Pediatric Infectious Diseases, University of California, Irvine, CA, USA; 12 Providence St. Joseph Health, Mission Viejo CA; 13 Al Azhar University, Cairo, Egypt; 14 Ascension Healthcare, Milwaukee, Wisconsin, USA; 15 Icahn School of Medicine at Mount Sinai, Department of Cardiology, NY, USA; 16 Arizona State University, School of Human Evolution and Social Change, Center for Evolution and Medicine, Arizona State University, Tempe, AZ, USA; 17 Institute for Advanced Study in Toulouse, France; 18 Saint Luke’s Mid America Heart Institute, Kansas City, Missouri, USA; 19 University of Missouri–Kansas City, USA; 20 MemorialCare, Southern California, USA; 21 Division of Cardiology, University of California, Irvine, Orange, California, USA; 22 Chapman University, Department of Health Economics and Anthropology, Economic Science Institute, Argyros School of Business and Economics, Orange, California, USA

## Abstract

**Background:**

Atrial fibrillation is the most common arrhythmia in post-industrialized populations. Older age, hypertension, obesity, chronic inflammation, and diabetes are significant atrial fibrillation risk factors, suggesting that modern urban environments may promote atrial fibrillation.

**Objective:**

Here we assess atrial fibrillation prevalence and incidence among tropical horticulturalists of the Bolivian Amazon with high levels of physical activity, a lean diet, and minimal coronary atherosclerosis, but also high infectious disease burden and associated inflammation.

**Methods:**

Between 2005–2019, 1314 Tsimane aged 40–94 years (52% female) and 534 Moseten Amerindians aged 40–89 years (50% female) underwent resting 12-lead electrocardiograms to assess atrial fibrillation prevalence. For atrial fibrillation incidence assessment, 1059 (81% of original sample) Tsimane and 310 Moseten (58%) underwent additional ECGs (mean time to follow up 7.0, 1.8 years, respectively).

**Findings:**

Only one (male) of 1314 Tsimane (0.076%) and one (male) of 534 Moseten (0.187%) demonstrated atrial fibrillation at baseline. There was one new (female) Tsimane case in 7395 risk years for the 1059 participants with >1 ECG (incidence rate = 0.14 per 1,000 risk years). No new cases were detected among Moseten, based on 542 risk years.

**Conclusion:**

Tsimane and Moseten show the lowest levels of atrial fibrillation ever reported, 1/20 to ~1/6 of rates in high-income countries. These findings provide additional evidence that a subsistence lifestyle with high levels of physical activity, and a diet low in processed carbohydrates and fat is cardioprotective, despite frequent infection-induced inflammation. Findings suggest that atrial fibrillation is a modifiable lifestyle disease rather than an inevitable feature of cardiovascular aging.

## Introduction

Atrial fibrillation (AF) is the most common arrhythmia in the post-industrialized world [1,2]. It is associated with a five-fold increase in the risk of stroke [3], which is the second leading cause of death in many industrialized nations [4]. AF is also associated with a three-fold increase in risk of heart failure and two-fold increases in risk of dementia and all-cause mortality [3,4]. The incremental cost of atrial fibrillation to the health care system has been estimated at 6–26 billion dollars per year in the United States alone [5].

AF prevalence is increasing worldwide due to its greater incidence among older adults and the aging of the global population [6,7]. AF prevalence in the U.S. is expected to increase up to 45% by 2030, to over 12 million cases [8], and to 14–17 million cases across Europe [9]. The increasing prevalence of potentially modifiable AF risk factors contributes to its growing prevalence [6]. AF risk factors include older age, hypertension, congestive heart failure, diabetes, peripheral vascular and coronary artery disease, valvular heart disease, alcohol excess, sleep apnea, systemic inflammation, male sex, and other cardiovascular disease risk factors (e.g. low high-density lipoprotein, HDL) [3,4,6,10]. Correlates of AF observed on a 12-lead electrocardiogram (ECG) include first-degree atrioventricular blocks, increased QRS duration, and bundle branch blocks [11].

Given the widespread and increasing prevalence of AF in post-industrialized populations, and its detection worldwide [12], AF has been considered a natural consequence of cardiovascular aging [13]. However, most estimates to date have been derived from high-income countries with more sedentary lifestyles. Prevalence and incidence rates appear to be generally lower in low-income countries, although country-level differences are not well explained by development status [12]. Thus, the relative roles of lifestyle and the biology of aging in AF incidence across diverse environments remain unclear.

### Study Goals and Hypotheses

To assess the extent to which lifestyle factors influence AF risk, we evaluate AF prevalence and incidence in the Tsimane and Moseten, two subsistence-based populations of lowland Bolivia with active lifestyles [14]. The Tsimane are an indigenous population of approximately 17,000 who fish, hunt, farm with hand tools, and forage for their food; they have minimal access to electricity, clean water, and public sanitation [15]. The Moseten (population ~3,000) are a genetically and ethno-linguistically related Bolivian population of horticulturalists who live in close proximity to Tsimane but who began acculturation into broader Bolivian society decades earlier [16]. The Tsimane mostly inhabit the Maniqui River basin area in the municipality of San Borja, while the Moseten territory is adjacent but closer to the Andes foothills west of the Tsimane area in the municipality of Palos Blancos. There is limited inter-marriage between Tsimane and Moseten, as their territories do not overlap. While Tsimane are largely endogamous, the Moseten in recent decades have intermarried with highland migrants to the region. Working with the Tsimane and Moseten to assess AF provides a rare opportunity to evaluate the extent to which lifestyle and environment might effectively reduce AF, beyond that of healthy subjects in industrialized nations. Tsimane have the lowest levels of coronary atherosclerosis ever observed, based on non-contrast X-ray computed tomography (CT) to evaluate coronary artery calcification [17]. Tsimane exhibit few other atherosclerotic risk factors, including hypertension [18], obesity, type 2 diabetes, and hyperlipidemia [17,19]. Tsimane diet is rich in fiber, polyunsaturated fatty acids, potassium, magnesium, and selenium [16], and low in saturated fat and preservatives. They have high physical activity levels beginning early in life [14].Though endurance-trained athletes show evidence of increased frequency of paroxysmal AF [20], the Tsimane do not typically engage in very high levels of vigorous activity. Instead, the Tsimane engage in high levels of low and moderate activity, and thus are rarely sedentary, throughout the day [14]. They show no evidence of lower extremity peripheral atherosclerosis as assessed by Ankle-Brachial Indices [21]. However, two AF risk factors that are prevalent among Tsimane include elevated levels of systemic inflammation, and low levels of high-density lipoproteins (HDL) [17,21]. Inflammation has been associated with increased AF prevalence in numerous studies [22,23,24], and the Tsimane experience frequent infections, which increases systemic inflammation and immune activation throughout life [25,26]. Low HDL is also a significant AF risk factor [27], and over half of Tsimane adults age 40+ years show HDL levels < 40 mg/dL [17].

Compared to Tsimane, Moseten lifestyle is more acculturated: Moseten have more schooling, have greater access to clean water and electricity, and many no longer speak the indigenous Moseten language but instead are monolingual Spanish speakers. Moseten also engage more often in cash cropping than Tsimane and have greater access to labor-saving technology, like chainsaws. Likely as a result of these differences, the Moseten show higher rates of obesity and type 2 diabetes, and have more processed foods and additives in their diet [16]. Nevertheless, compared to populations in high-income countries, Moseten lifestyle as subsistence farmers is still closer to that of the horticultural-forager Tsimane in terms of physical activity level and other AF risk factors. Investigating Moseten AF thus represents an opportunity to explore the role of lifestyle change on AF in a genetically related population inhabiting a similar rural environment within Bolivia.

Our aims are to (1) assess risk factors for AF among Tsimane and Moseten; and (2) estimate their AF prevalence and incidence and compare against other world populations.

## Methods

### Risk factors

Baseline demographic information was collected by a mobile biomedical team along with measurements of the following risk factors: height, weight, waist circumference, blood lipids (total cholesterol, LDL, HDL and triglycerides) from fasting morning samples, inflammatory markers (high sensitivity C-reactive protein [hs-CRP], erythrocyte sedimentation rate [ESR], interleukin-6 [IL6]), and systolic and diastolic blood pressure (see 17, 18 for detailed procedures) [17,18]. Hypertension and diabetes were classified according to clinical guidelines [28].

### AF Prevalence and Incidence

Trained Bolivian physicians (RQG, ECL, DER) conducted a resting standard 12-lead electrocardiogram (ECG). Tsimane ECGs were reviewed by two cardiologists (CJR and KT) blinded to subject information and independently evaluated by three cardiologists (GST, MIM, LSW). Moseten ECGs were reviewed by one of the same cardiologists (CJR) and an additional cardiologist (MAE). ECGs were coded as either positive or negative for AF. There was no disagreement in diagnoses among coders. ECG measures of PR and QRS were also recorded.

To assess AF prevalence, at least one ECG was performed from 2005–2019 on 1314 Tsimane aged 40+ years, which at the time represented 70% of the adult population in that age range (***Table 1***; Figure S1 for STROBE sample flow chart). AF was defined as in the 2006 ACC/AHA/ESC guidelines [29]. Participants also underwent routine medical screening by a mobile medical team consisting of internists, medical technicians, and biochemists. A patient’s first ECG was used to assess AF prevalence, and 81% (n = 1059) of the sample underwent at least one subsequent ECG to assess incidence. Between 2015 to 2018, one ECG was performed on 534 Moseten aged 40+ years by the same team and using the same protocol, covering 95% of the eligible population aged 40+. A second ECG was collected among 310 (58%) of these Moseten participants (***Table 2***, Figure S2).

**Table 1:** **Tsimane sample composition and distribution of AF risk factors.** Standard deviations and standard errors are given in parentheses (for means and proportions, respectively). Significance column provides *p*-values for testing differences across age groups based on ANOVA.


AGE GROUP (YEARS)	N	39–49	50–59	60–69	70–79	≥ 80	TOTAL	SIGNIFICANCE

N with baseline ECG		670	328	204	90	22	1314	

Proportion Male		0.52	0.51	0.55	0.49	0.36	0.52	

**Anthropometric measures**								

Height cm	1307	157.0 (7.2)	155.8 (7.7)	155.1 (7.4)	151.6 (7.9)	149.9 (6.2)	155.9 (7.5)	<0.001

Weight kg	1307	60.4 (9.6)	58.4 (10.3)	56.3 (9.4)	53.2 (8.7)	47.8 (7.9)	58.5 (10.0)	<0.001

BMI kg/m^2^	1306	24.4 (3.1)	24.0 (3.6)	23.3 (3.1)	23.1 (3.4)	21.2 (2.2)	24.0 (3.3)	<0.001

% BMI > 30 kg/m^2^	1306	5.0 (0.8)	5.2 (1.2)	3.5 (1.3)	4.4 (2.2)	0.0 (0.0)	4.7 (0.6)	0.362

Waist Circ. in	1049	34.9 (3.3)	35.0 (4.1)	35.2 (3.7)	35.6 (4.0)	33.5 (2.4)	35.0 (3.6)	0.732

% Waist Circ. High	1049	24.6 (1.9)	26.0 (2.7)	31.1 (3.7)	25.0 (5.3)	5.9 (5.9)	25.6 (1.4)	0.938

**Lipid profiles**								

Total Cholesterol mg/dL	1112	142.9 (27.6)	146.8 (30.6)	140.9 (26.9)	137.8 (27.6)	139.6 (26.5)	143.3 (28.4)	0.064

% Cholesterol > 240 mg/dL	1112	0.35 (0.25)	0.68 (0.48)	0.0 (0.0)	0.0 (0.0)	0.0 (0.0)	0.36 (0.18)	0.498

LDL mg/dL	1032	86.6 (25.7)	89.7 (27.9)	85.0 (25.6)	80.3 (25.3)	82.5 (21.7)	86.7 (26.2)	0.045

% LDL > 130 mg/dL	1032	5.28 (0.97)	6.77 (1.54)	4.52 (1.67)	5.97 (2.92)	0.00 (0.00)	5.52 (0.71)	0.714

HDL mg/dL	1035	37.2 (7.5)	38.0 (6.7)	37.1 (6.4)	38.0 (7.6)	37.1 (9.6)	37.5 (7.2)	0.045

Triglycerides mg/dL	1117	109.0 (46.9)	113.9 (52.7)	106.1 (45.9)	105.9 (41.0)	105.6 (22.6)	109.6 (47.8)	0.519

% Triglycerides > 200 mg/dL	1117	4.20 (0.84)	4.76 (1.24)	4.29 (1.59)	1.37 (1.37)	0.00 (0.00)	4.12 (0.59)	0.289

**Inflammatory Markers**								

hs-CRP mg/dL	758	2.4 (1.8)	2.6 (2.3)	3.1 (2.3)	2.3 (1.6)	4.4 (2.7)	2.6 (2.0)	<0.001

% hs-CRP > 3 mg/dL	758	37.0 (2.5)	42.9 (3.4)	53.8 (4.6)	45.5 (7.6)	66.7 (14.2)	42.2 (1.8)	<0.001

ESR mm/h	1304	27.1 (15.1)	30.3 (15.1)	38.8 (20.9)	43.7 (22.3)	58.4 (19.0)	31.4 (17.9)	<0.001

% Elevated ESR	1304	45.6 (1.9)	59.3 (2.7)	72.3 (3.2)	76.4 (4.5)	95.5 (4.6)	56.1 (1.4)	<0.001

IL6 (pm/mL)	740	1.6 (0.7)	1.6 (0.5)	1.6 (0.9)	1.6 (1.6)	1.5 (1.3)	1.6 (0.8)	<0.001

**Blood Pressure and Hypertension**								

Heart Rate (BPM)	1314	51.1 (26.0)	48.1 (27.6)	49.2 (28.4)	55.9 (29.5)	57.6 (26.4)	50.5 (27.1)	0.640

Systolic Blood Pressure mmHg	1305	111.1 (8.0)	114.4 (10.2)	118.0 (12.7)	121.9 (16.4)	121.5 (24.0)	113.9 (11.1)	<0.001

% SBP > 120 mmHg	1305	11.3 (1.2)	19.9 (2.2)	34.2 (3.4)	45.6 (5.3)	38.1 (10.9)	19.8 (1.1)	<0.001

Diastolic Blood Pressure mmHg	1305	69.7 (5.8)	70.8 (6.1)	71.6 (7.9)	70.8 (7.8)	68.2 (9.8)	70.3 (6.5)	0.004

% DBP > 80 mmHg	1305	3.3 (0.7)	6.8 (1.4)	11.4 (2.2)	7.8 (2.8)	4.8 (4.8)	5.8 (0.6)	0.002

% Hypertensive (>130/80 mmHg)	1305	3.8 (0.7)	8.9 (1.6)	20.3 (2.8)	26.7 (4.7)	9.5 (6.6)	9.3 (0.8)	<0.001

**ECG Measures**								

PR ms	1312	162 (19)	162 (19)	162 (18)	162 (20)	160 (21)	162 (19)	0.990

% PR > 200 ms	1312	1.79 (0.5)	2.44 (0.9)	2.46 (1.1)	3.37 (1.9)	4.56 (4.6)	2.21 (0.4)	0.328

QRS ms	1313	103 (12)	103 (12)	104 (17)	104 (18)	99 (14)	103 (14)	0.999

% QRS > 120 ms	1313	7.6 (1.0)	5.8 (1.3)	10.3 (2.1)	10.0 (3.2)	4.6 (4.6)	7.7 (0.7)	0.316

**Coronary Calcium scores from CT scan**								

% CAC > 100 AU	823	0.5 (0.38)	3.5 (1.2)	8.5 (2.34)	4.4 (3.1)	30.8 (13.3)	3.5 (0.6)	<0.001


*Note*: Sample sizes vary, especially for serum biomarker analyses, due to logistical complications in the field (e.g., inability to store serum). CT sample sizes are smaller because of stratified sampling for ages 40–54.

To assess Tsimane incidence, all follow-up ECGs (n = 1059 individuals totaling 7395 risk years) were examined with an average time to follow-up of 7.0 years (***Table 3***). Moseten incidence is based on a smaller follow-up sample (n = 310 totaling 542 risk years; average time to follow-up = 1.8 years). Tsimane and Moseten adults age 40+ years not sampled in the prevalence phase or missed during the incidence phase show minimal differences in AF risk factors (Supplementary Table S1).

### Verbal Autopsies

We recognize that our estimates of AF prevalence and incidence may be biased due to potentially higher case fatality rates, given the limited medical treatment in the region. To investigate this possibility, one THLHP physician (RQG) and Tsimane assistant conducted verbal autopsies for all Tsimane deaths occurring between October 2008 and December 2013 (n = 209 deaths) using the 2012 World Health Organization Verbal Autopsy Instrument [30].

### Statistical Analysis

Crude prevalences were determined by dividing the number of cases diagnosed with AF by the total number of participants undergoing their initial ECG. Crude incidence rates were calculated by dividing the total number of new cases diagnosed from the follow-up ECG by the number of risk years between baseline and last ECG (if all were negative) or between the negative and positive ECGs (if a new incidence occurred).

**Figure 1:**
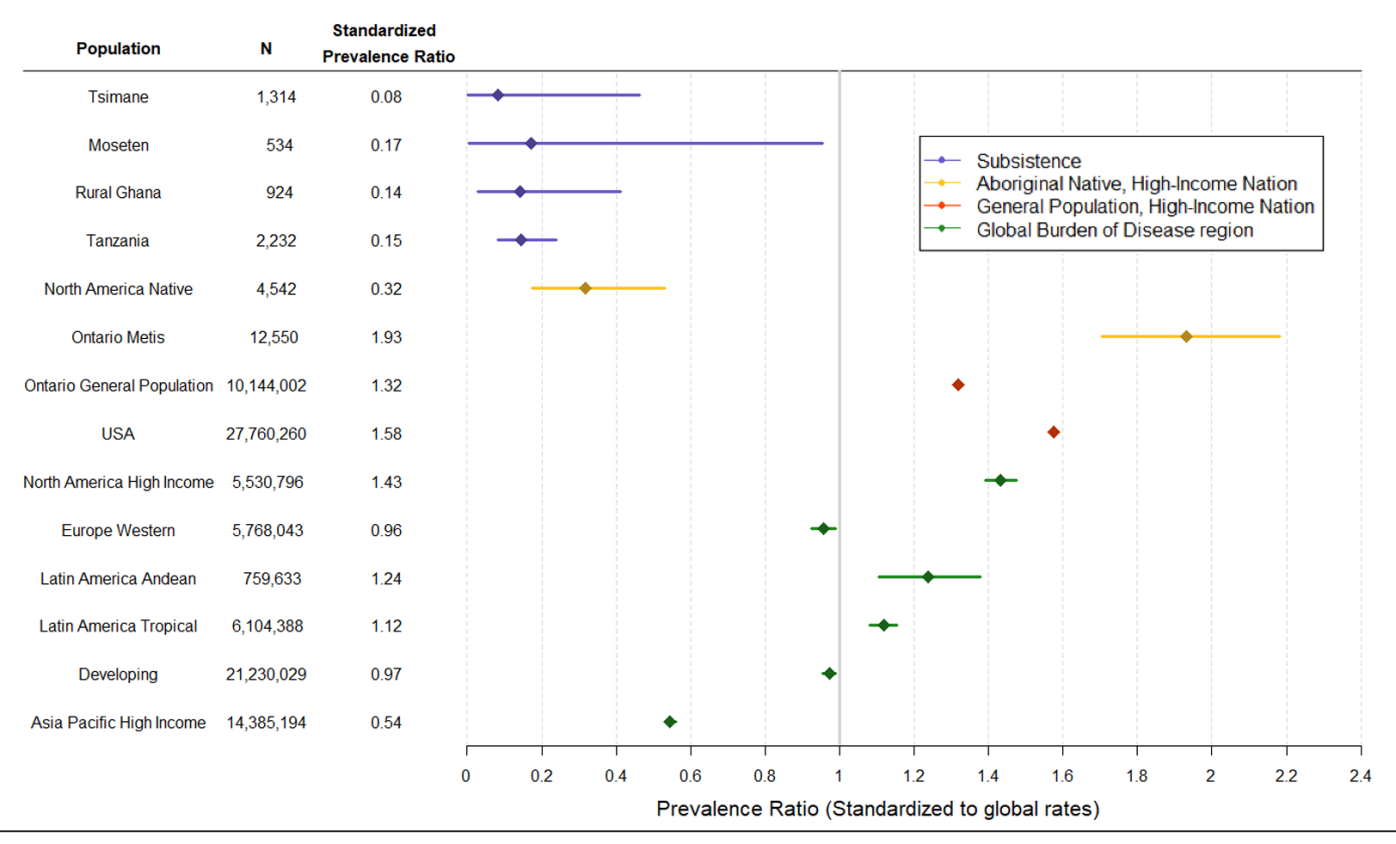
Age-standardized atrial fibrillation prevalence ratios, with 95% CIs. Ratios compare the observed prevalence in each population with the expected prevalence if that population had the average age-specific AF prevalences of a global GBD sample (2010 total prevalence of 1366/100,000), as reported by Chugh et al [12].

To compare AF prevalence across populations, we selected papers reporting AF prevalence in rural subsistence-level samples (Ghana and Tanzania) [31,32] and among indigenous groups living in industrialized nations (Indigenous Australians and Native Americans) [33,34,35,36]. We also selected studies in industrialized populations that were population representative, used similar methodology for AF diagnosis, and provided age-specific AF rates so that age-standardized rates could be calculated [37,38,39,40]. Lastly, we included recently published AF prevalences for Global Burden of Disease (GBD) world regions based on 184 studies, that used similar inclusion and exclusion criteria, and so may also include atrial flutter and paroxysmal AF [12]. We employ indirect standardization using age-specific AF prevalences from the GBD sample to compute comparable estimates of AF prevalence across populations (***Figure 1***). This was done to account for the different age ranges among samples, which prevented us from directly standardizing the age-specific prevalences of each sample to a standard population structure.

### Ethics Statement

All phases of the study were approved by the Institutional Review Boards of the University of New Mexico Health Sciences Center and the University of California, Santa Barbara. Additionally, the Tsimane government (Gran Consejo Tsimane), village leaders, and study participants approved all protocols.

## Results

### AF Risk Factors

Tsimane and Moseten adults are short in stature (mean = 156 and 155 cm, respectively) with a corresponding low body weight (mean ~59 and 62 kg, respectively) (***Tables 1, 2***). Mean body mass index (BMI) for Tsimane is 24 kg/m^2^ and 5% of adults are obese (BMI ≥ 30 kg/m^2^). Moseten show larger mean BMI (26.0 kg/m^2^) and higher obesity prevalence (18%).

Tsimane total fasting cholesterol levels are low (mean = 143 mg/dL, and < 1% have an elevated value ≥ 240 mg/dL. Low-density lipoproteins (LDLs) and triglycerides are also low (mean = 87 and 110 mg/dL, respectively). Only 5.5% had LDL ≥ 130 mg/dL and 4% had triglycerides ≥ 200 mg/dL. HDL values were also low (mean = 37.5 mg/dL). Moseten show higher levels of most blood lipids: mean total cholesterol is 159 mg/dL (3% with levels ≥ 240 mg/dL), mean LDL is 106 (22% ≥ 130 mg/dL), mean triglycerides are 132 mg/dL (14% ≥ 200 mg/dL). Only Moseten HDLs (mean = 37.6 mg/dL) are similar to Tsimane levels.

Inflammatory markers are elevated among both Tsimane and Moseten. Median levels of hs-CRP are 2.6 mg/dL for both populations, and nearly half of adults show values > 3.0 mg/dL. ESR is also high and increases with age in both populations: 56% of Tsimane and 58% of Moseten show elevated ESR levels. Interleukin-6 (IL-6) levels are also high, especially at older ages among Moseten (***Table 2***). Blood pressure (BP) among the Tsimane participants is low, with mean systolic and diastolic BP being 114 mmHg and 70 mmHg, respectively (see also Gurven, Blackwell, Eid, Stieglitz, and Kaplan) [18]. Tsimane hypertension (> 130 mmHg systolic and/or 80 mmHg diastolic) is minimal (9% prevalence) compared to Moseten (37% prevalence).

**Table 2:** **Moseten sample composition and distribution of AF risk factors.** Standard deviations are given in parentheses (for means), standard error (for proportions). “Total” column provides sample means age-standardized to Tsimane sample age distribution. Significance column provides *p*-values for testing differences across age groups based on ANOVA.


AGE GROUP (YEARS)	N	40–49	50–59	60–69	70–79	≥ 80	TOTAL (AGE-ADJUSTED)	SIGNIFICANCE

N with baseline ECG		208	155	121	39	10	534	

Proportion Male		0.47	0.5	0.54	0.62	0.4	0.5	

**Anthropometric Measures**								

Height cm	527	156.1 (7.1)	154.8 (6.7)	153.3 (8.0)	151.8 (9.5)	150.6 (7.1)	154.8 (7.4)	<0.001

Weight kg	523	64.5 (12.4)	62.6 (11.1)	58.2 (13.0)	58.2 (15.0)	60.5 (9.0)	62.4 (12.4)	<0.001

BMI kg/m^2^	523	26.5 (5.1)	26.1 (4.2)	24.6 (4.7)	25.1 (5.3)	26.6 (3.3)	26.0 (4.8)	0.002

% BMI > 30 kg/m^2^	523	21.2 (2.9)	18.2 (3.1)	10.2 (2.8)	21.1 (6.7)	10.0 (10.0)	18.3 (1.7)	0.088

**Lipid Profile**								

Total Cholesterol mg/dL	368	157.9 (30.1)	162.4 (37.8)	157.2 (35.2)	161.9 (24.9)	158.3 (34.5)	159.2 (33.0)	0.873

% Cholesterol > 240 mg/dL	368	2.9 (1.4)	4.5 (2.0)	2.3 (1.6)	0.0 (0.0)	0.0 (0.0)	3.0 (0.9)	0.28

LDL mg/dL	363	106.3 (35.7)	108.8 (37.9)	99.9 (35.8)	107.3 (27.4)	106.3 (30.3)	105.9 (35.7)	0.286

% LDL > 130 mg/dL	363	19.9 (3.4)	24.6 (4.1)	19.3 (4.2)	26.1 (9.4)	33.3 (21.1)	21.7 (2.2)	0.991

HDL mg/dL	363	37.6 (9.1)	36.5 (9.1)	39.2 (9.3)	37.8 (9.1)	35.1 (3.4)	37.6 (9.1)	0.193

Triglycerides mg/dL	365	137.9 (74.6)	134.2 (81.1)	113.0 (59.8)	135.0 (71.0)	103.1 (25.5)	131.8 (73.2)	0.047

% Triglycerides > 200 mg/dL	365	15.4 (3.1)	15.3 (3.4)	10.2 (3.3)	16.7 (7.8)	0.00 (0.00)	14.3 (1.9)	0.431

**Inflammatory Markers**								

hs-CRP mg/dL	109	2.6 (2.7)	2.5 (2.1)	2.9 (3.0)	3.5 (1.9)	7.3 (0.0)	2.6 (2.7)	0.315

% hs-CRP > 3 mg/dL	109	47.4 (8.2)	42.1 (8.1)	50.0 (9.3)	50.0 (50.0)	100.0 (NA)	47.6 (5.9)	0.275

ESR mm/h	449	29.6 (17.3)	33.8 (20.4)	38.5 (22.0)	46.9 (24.1)	39.0 (21.3)	33.6 (19.6)	<0.001

% Elevated ESR	449	49.7 (3.9)	60.2 (4.3)	64.4 (4.7)	84.9 (6.3)	70.0 (15.3)	57.8 (2.4)	<0.001

IL6 (pm/mL)	114	1.6 (0.3)	1.6 (1.1)	1.6 (0.9)	7.8 (8.8)	13.3 (0.0)	1.6 (1.1)	0.259

**Blood Pressure and Hypertension**								

Heart Rate (BPM)	533	64.0 (9.0)	65.3 (9.9)	64.4 (9.2)	69.5 (16.1)	67.8 (10.1)	64.9 (10.0)	0.019

Systolic Blood Pressure mmHg	526	116.9 (12.6)	118.9 (15.9)	125.0 (15.1)	130.8 (16.5)	144.8 (25.3)	120.3 (14.5)	<0.001

SBP > 120 mmHg	526	35.8 (3.4)	39.2 (4.0)	55.4 (4.5)	71.1 (7.5)	90.0 (10.0)	43.5 (2.1)	<0.001

Diastolic Blood Pressure mmHg	527	74.9 (10.1)	76.8 (11.6)	78.9 (10.2)	81.5 (9.3)	92.2 (15.2)	76.9 (10.6)	<0.001

% DBP > 80 mmHg	527	26.5 (3.1)	33.1 (3.8)	39.7 (4.5)	47.4 (8.2)	80.0 (13.3)	32.9 (2.0)	<0.001

% Hypertensive (>130/80 mmHg)	527	29.4 (3.2)	37.0 (3.9)	45.5 (4.6)	55.3 (8.2)	100.0 (0.0)	37.3 (2.1)	<0.001

**ECG Measures**								

PR ms	528	150 (19)	152 (20)	153 (20)	145 (21)	158 (33)	151 (20)	0.937

% PR > 200 ms	528	1.5 (0.8)	2.0 (1.1)	1.7 (1.2)	0.0 (0.0)	11.1 (11.1)	1.7 (0.6)	0.565

QRS ms	533	104 (13)	102 (10)	106 (17)	104 (15)	96 (8)	104 (13)	0.872

% QRS > 120 ms	533	4.3 (1.4)	4.5 (1.7)	10.7 (2.8)	15.4 (5.9)	0.0 (0.0)	6.2 (1.0)	0.018

Prevalence of first-degree block (PR > 200 ms) was rare in both Tsimane and Moseten populations (2%). Across both populations, 6–8% had QRS > 120 ms. Resting heart rates are relatively low, averaging 50.5 and 64.9 beats per minute among Tsimane and Moseten, respectively.

Coronary artery disease as assessed by non-contrast CT scan of coronary calcium (CAC) is largely absent among Tsimane. Mean CAC values range from < 2 Agaston units (AU) for adults aged 40–49 to 68 AU for adults aged 80+ years. Overall, only 3.5% of Tsimane aged 40+ years have a CAC score over 100 AU (considered moderate risk) [17]. Analysis of CAC among Moseten is currently underway.

Only a minority of Tsimane and Moseten, mostly men, smoked regularly. The majority of people who did smoke only smoked a few cigarettes per month. We estimate that the average Tsimane ‘smoker’ smoked < 0.3 pack years in their lifetime. Smoking is more common among Moseten: 39% reported smoking regularly, and the average Moseten smoker smoked 2.6 pack years in their lifetime.

## AF Prevalence and Incidence

### Tsimane

Only one of 1314 ECGs demonstrated AF (***Table 2***), yielding a crude prevalence rate of 0.076%, or < 1 person per 1,000 (***Table 3***). There was one new AF case in 7395 risk years from 1059 adults with > 1 ECG. This represents a crude incidence rate of 0.14 per 1,000 risk years.

**Table 3:** Crude prevalence and incidence of atrial fibrillation.


AGE GROUP (YEARS)	40–49	50–59	60–69	70–79	≥ 80	TOTAL

**TSIMANE**						

Number of individuals	670	328	204	90	22	1314

Number of AF cases	0	0	0	1	0	1

**AF prevalence** (per 1,000)	0	0	0	11.11	0	0.76

N with >1 ECG	541	286	157	60	15	1059

Total Person-Years	3726	2190	1074	335	70	7395

Average Risk Years per Person	6.89	7.66	6.84	5.59	4.67	6.98

Number of new AF cases	0	0	0	0	1	1

**AF incidence** (per 1,000 person years)	0	0	0	0	14.29	0.14

**MOSETEN**						

Number of individuals	208	156	121	39	10	534

Number of AF cases	0	0	0	1	0	1

**AF prevalence** (per 1,000)	0	0	0	25.64	0	1.87

N with > 1 ECG	96	92	88	25	9	310

Total Person-Years	170	162	153	43	14	542

Average Risk Years per Person	1.78	1.76	1.74	1.73	1.55	1.75

Number of new AF cases	0	0	0	0	0	0

**AF incidence** (per 1,000 person years)	0	0	0	0	0	0

#### Moseten

Only one of 534 ECGs demonstrated AF (***Table 2***), representing a crude prevalence of 0.187% (1.87 per 1,000). There were no new AF cases in 542 risk years from 311 adults with > 1 ECG (***Table 3***). Despite observing only one case of AF, we report three observations of premature atrial contractions, which are believed to play a critical role in AF pathogenesis in other populations [41,42]. No cases of atrial flutter were observed among either the Moseten or Tsimane.

#### Clinical Characteristics of All Three AF Cases

***Case 1 (observed in Tsimane prevalence phase):*** The only person with AF on his first ECG was a 70-year-old man in 2010. At last contact with the study in 2019, he was 79 years of age. Echocardiography demonstrated no valvular or structural heart disease and a normal ejection fraction. No other obvious AF risk factors were observed (BMI = 23 kg/m^2^, BP: 100/65 mmHg, total cholesterol = 137 mg/dL, LDL = 77 mg/dL, HbA1c = 5.6%). He has had five ECGs conducted by our project physicians from 2010 to 2019, and AF was observed in all of them, consistent with permanent AF.

***Case 2 (observed in Tsimane incidence phase):*** The only adult who developed AF in the incidence phase was an 85-year-old woman whose first medical contact with our project was in 2010. She underwent a standard medical evaluation by a project physician, including an ECG and echocardiogram which demonstrated moderate aortic stenosis. She again received medical attention in August of 2016 for evaluation of an undiagnosed illness. Repeat echocardiography showed severe aortic stenosis and new AF based on the ECG. Three days later she went into pulseless electrical activity and died from end stage aortic stenosis; both prior ECGs showed normal sinus rhythm without left ventricular hypertrophy. Prior observations also showed no obvious AF risk factors (BMI = 20.1 kg/m^2^, BP: 100/60 mmHg, pulse: 68 bpm, total cholesterol = 94.9 mg/dL, LDL = 45.2 mg/dL).

***Case 3 (observed in Moseten prevalence phase):*** The only resident of a Moseten community with AF was a 72-year-old man. Though he resides in a Moseten village, he and his family migrated there from the Bolivian highlands and he is not of Moseten descent. AF was confirmed on two visits separated by two years (July 2016, July 2018), suggestive of long-standing persistent AF. Echocardiography demonstrated mild to moderate aortic stenosis in 2016. He was obese (BMI = 40.9 kg/m^2^, total body fat percentage = 38%), diabetic (fasting blood glucose = 241 mg/dL in 2016, and was 122 mg/dL in 2018 after treatment), hypertensive (110/97 mmHg), had elevated resting heart rate (> 90 bpm on multiple occasions), but had low cholesterol (total cholesterol =132 mg/dL, LDL=70.4 mg/dL).

*Mortality Selection:* One possibility for low AF prevalence and incidence is that the Tsimane or Moseten might experience a high AF case fatality rate given limited treatment options. However, our verbal autopsies revealed at most only two suspected cases where deaths could potentially be attributed to undiagnosed AF: an 88-year-old woman who died of a possible stroke, and a 75-year-old man who died of an apparent myocardial infarction. Both of these individuals were in our ECG sample but had normal rhythm without conduction defects and no evidence of significant pathology prior to their death. It is therefore unlikely that mortality selection could account for the low AF prevalence and incidence rates we report here.

### Tsimane and Moseten AF in Comparative Perspective

In order to compare the Tsimane prevalence of AF to other populations, ***Figure 1*** uses indirect standardization to compute age-standardized risk ratios of observed AF prevalence in diverse populations relative to the expected prevalence in those same populations if they had the average age-specific AF prevalences of a 2010 global reference sample from the Global Burden of Disease study of AF [12]. The first cluster (in blue) represents four subsistence populations: the Tsimane, Moseten, and rural samples from Ghana and Tanzania [31,32]. Prevalence rates across all four subsistence populations are exceedingly low, approximately 0.08–0.17 (1/12–1/6 as high as the global average), with overlapping confidence intervals.

The second cluster represents aboriginal and native populations in the U.S., Canada and Australia, who no longer practice a subsistence-based lifestyle. While one Native American sample from the Strong Heart Study [33] shows a relatively low prevalence ratio, about 1/3 of the global reference, it is still 2–4 times higher than observed in subsistence populations. Two additional studies report higher AF prevalence among Native Americans. Ontario Métis natives show highly elevated prevalence (~50% higher) compared to non-native Canadians [35,36,43]. A larger sample of Native Americans from the Veteran’s Administration in 1999, though restricted to men, shows a much higher prevalence among Native Americans than what we report in ***Figure 1***, similar to whites in that study [34]. More recent data show Native Americans of California to have higher incident AF than all other U.S. ethnic groups, attributed in part to excess chronic kidney disease and diabetes [44]. Australian Aborigines living in urban areas exhibit highly elevated AF prevalence, compared to the subsistence populations and global reference analyzed here, though this Aboriginal sample comes from urban hospital admissions over a 10-year period (and hence is not shown in ***Figure 1***) [36]. In that study, Aborigines showed higher AF prevalence at younger ages (< 60 y) and lower AF prevalence at older ages (> 70 y) than non-indigenous Australians.

As a regional comparison, we include tropical and Andean regions of Latin America. Both regions show rates 12–24% higher than the global reference. Western Europe and the GBD designation for ‘developing’ countries show similar AF prevalences as the global reference. North American AF prevalence is 43% higher, and the U.S. specifically is 58% higher, than the global reference. The Asia Pacific region shows the lowest AF prevalence for all post-industrialized countries, at 54% of the global reference.

## Discussion

AF is exceedingly rare among Tsimane and Moseten Amerindians of Bolivia: we found just one AF case in the prevalence phase of each population and one case in the incidence phase of the Tsimane. AF prevalence was therefore 8–18% that of Western Europe, and 5–11% that of the United States. It is also 15–31% that of Asia Pacific, which is the GBD world region with the lowest rates of AF [12]. The combined crude incidence rate of AF for Tsimane and Moseten is 0.13 per thousand risk years.

Tsimane and Moseten exhibit minimal AF despite having several traditional risk factors, including elevated inflammatory biomarkers (e.g., CRP, IL-6, ESR [22]), and low HDL. The fact that AF is almost absent among Tsimane and Moseten suggests that the source of inflammation itself may play a role in AF etiology [17,26]. In rural Bolivia, most inflammation comes from diverse infections (bacterial, parasitic, viral) that afflict individuals from a young age, while among many sedentary industrialized populations, the combination of obesity, cigarette smoking, air pollution, and other factors promotes chronic low-grade inflammation [45]. Given the heavy chronic infectious burden amongst the Tsimane and Moseten, and potential links between pathogen burden and the development of AF [46], one would expect to have found more cases of AF than observed. This interesting finding calls into question the particular inflammatory pathways that lead to AF. In industrialized populations, low HDL is also considered a moderate risk factor for AF [27]; this does not appear to be the case for Tsimane and Moseten, where over half of all individuals have low HDL but also low LDL and negligible AF risk.

AF has an exceptionally low prevalence in the four subsistence populations where it has been measured (***Figure 1***) [31,32]. While many traditional AF risk factors were low in these populations, the two African populations showed higher levels of hypertension, suggesting that hypertension alone is insufficient to increase AF risk. Despite being the most common medical condition associated with AF worldwide [6], hypertension likely must interact with other cardiovascular disease risk factors like dyslipidemia, diabetes, and sedentary lifestyle to increase AF susceptibility. Low AF risk among the two African populations, and in sub-Saharan African countries more generally, are also noteworthy given high reported stroke incidences in the region [47]. AF patients in sub-Saharan Africa also tend to be younger than in other world regions, and experience high case fatality and rheumatic valvular heart disease [48]. AF incidence is expected to increase substantially in sub-Saharan Africa over the next few decades [12,48], highlighting the need for more research on AF etiology in the region. The Tsimane and Moseten also exhibit greater systemic inflammation and lower HDL than other subsistence populations, but this does not appear to lead to greater AF prevalence. These observations in subsistence populations suggest that multiple risk factors in concert may be necessary to increase AF risk.

Despite the fact that Moseten lead a more modernized lifestyle than Tsimane, they do not manifest significantly higher AF prevalence or incidence. As most lifestyle differences among Moseten have occurred in the past three decades, it is possible that older Moseten have largely had similar cumulative exposures as age-matched Tsimane. Our results therefore suggest that lifecourse, or perhaps particularly early life, exposures warrant consideration beyond biomarkers assessed in later adulthood. An expectation is that differences in AF incidence, and presumably CVD, diabetes, and related comorbidities, will increasingly manifest in younger adults over time.

### Limitations

In order to reduce measurement bias when assessing AF prevalence, we took steps to make sure that people who were not sampled (n = 68 individuals, 4.6% of the study population) were not missed due to illness or other comorbidities that could be associated with AF. However, people not sampled were likely among the healthiest, either traveling to other villages or locations during team visits, or too busy working that they declined medical attention (very few individuals, Figure S1, S2).

While mortality selection might explain why indigenous Australians have higher AF prevalence than non-indigenous Australians only before age 60 years [36], it does not explain the low levels of AF risk we report among Tsimane and Moseten. Consistent with what we report for verbal autopsies, an earlier study examining retrospective causes of death among Tsimane similarly reported minimal evidence of strokes or infarcts [49]. A study of stroke incidence among another Bolivian Amazonian population (Chiquitano) also reported very low incidence (174/100,000) over a one-year period [50].

Another potential weakness is that we only performed resting 12-lead ECGs, which sample 10 seconds at a time. With our methodology, it would be difficult to detect cases of intermittent or paroxysmal AF. It has been proposed that paroxysmal AF may be more common in some ethnicities than the persistent AF usually reported for Caucasians, especially among ethnicities with higher stroke risk [51]. Thus, it is possible that we and others using similar methods may be underestimating AF. Indeed, the use of higher-sensitivity detection methods has been shown to reduce ethnic differences in AF prevalence [52]. Nevertheless, the comparative data from other population-level studies relied on similar ECG methodology, and so our results are comparable to most studies reported in ***Figure 1***.

Lastly, we acknowledge that ~140 genetic loci affecting AF have been identified to date among participants mostly of European descent [53]. Genetic risk prediction of AF is still in its infancy, and the relevance of these loci and ancestry-specific rare variants in contributing to the low AF prevalence among Tsimane, Moseten, and other subsistence populations remains to be determined.

## Conclusion

Our findings of exceptionally low AF prevalence and incidence among rural Bolivian horticulturalists suggest that a subsistence lifestyle with high rates of physical activity and a diet relatively low in processed carbohydrates and harmful fats can be heart-healthy despite high levels of inflammation and low HDLs. Some lifestyle alterations associated with incipient modernization may be insufficient to increase AF risk, as suggested here by similar AF prevalences among Tsimane and Moseten. AF prevalence is higher among indigenous populations that are no longer living a traditional subsistence lifestyle, suggesting that combined changes in physical activity, diet, and other risk factors associated with marginalization, discrimination, and lifestyle disruption can increase vulnerability to AF, especially when coupled with low access to quality healthcare. Rather than being viewed as an inevitable feature of cardiovascular aging, it appears that AF is better viewed as a modifiable lifestyle disease with ample scope for prevention.

## Data Accessibility Statement

Code and anonymized, disguised data are provided to recreate ***Tables 1, 2, 3*** and Supplementary Table S1: *https://github.com/babeheim/tsimane-afib*. Undisguised but anonymous data, however, can be shared upon reasonable request to the corresponding author.

## Additional Files

The additional files for this article can be found as follows:

10.5334/aogh.3252.s1 Supplementary Figure S1. STROBE Diagram for Tsimane subject recruitment. N = 1871 refers to all Tsimane who were at least age 40 years from 2005–2019, considering that the THLHP medical team sampled adults age 40+ throughout the entire study period (i.e. not only at baseline). N = 1314 adults had at least one ECG. 557 adults were not sampled, either because ECGs were not measured on all medical rounds (most common, largely due to damaged equipment, lack of electricity, and in some cases lack of time), or because adults were either not present in the village during visits when ECGs assessed (common reason), or were present in the community but did not show up for their clinical visit (less common). 255 adults were lost to follow-up, due either to not being in the village during follow-up visits (most common), being present but not showing up for their clinical visit (less common), or being seen by project physicians but refusing an ECG (rare).

10.5334/aogh.3252.s2 Supplementary Figure S2. STROBE Diagram for Moseten subject recruitment. The prevalence phase is based on one medical round in 2015–2016; incidence phase is based on a follow-up visit in 2017–2018. Of the 561 eligible Moseten aged 40+ years, 20 were either not present in the community during THLHP visits (most common), or were present but did not visit our mobile clinic (less common). Loss to follow-up during the second visit was due to not revisiting all of the same villages seen during the prevalence phase, and slightly poorer attendance at our clinic.

10.5334/aogh.3252.s3 Supplementary Table S1. Potential sample bias in prevalence and incidence phases. Biomarker comparisons between those who received or did not receive an ECG during the prevalence phase, and those who did or did not receive a follow-up ECG in the incident phase. Mean values are compared using student t-tests. Blue color indicates lower AF risk among unsampled adults, whereas red color indicates higher AF risk. In the prevalence phase, unsampled Tsimane and Moseten are similar or show lower AF risk than those sampled with an ECG. In the incidence phase, Tsimane from the prevalence phase who were lost to follow-up ECG showed only slightly higher AF risk than those sampled (2.1 years older, 2.6 mg/dL lower HDL, 1.8 mmHg higher systolic blood pressure, 6.5 mm/hr higher ESR), though also show lower AF risk for LDL (14 mg/dL lower) and total cholesterol (13.3 mg/dL lower). Among Moseten, those lost to follow-up showed only slightly higher BMI (0.9 kg/m^2^ higher).
