# Supplementary material for: Very Low Prevalence and Incidence of Atrial Fibrillation among Bolivian Forager-Farmers
Source: Ann Glob Health. 2021 Feb 16;87(1):18. doi: 10.5334/aogh.3252 (PMC7894370; doi:10.5334/aogh.3252)

Supplementary Figure S1. STROBE Diagram for Tsimane subject recruitment. N=1871 refers to all Tsimane who were at least age 40 years from 2005-2019, considering that the THLHP medical team sampled adults age 40+ throughout the entire study period (i.e. not only at baseline). N=1314 adults had at least one ECG. 557 adults were not sampled, either because ECGs were not measured on all medical rounds (most common, largely due to damaged equipment, lack of electricity, and in some cases lack of time), or because adults were either not present in the village during visits when ECGs assessed (common reason), or were present in the community but did not show up for their clinical visit (less common). 255 adults were lost to follow-up, due either to not being in the village during follow-up visits (most common), being present but not showing up for their clinical visit (less common), or being seen by project physicians but refusing an ECG (rare).

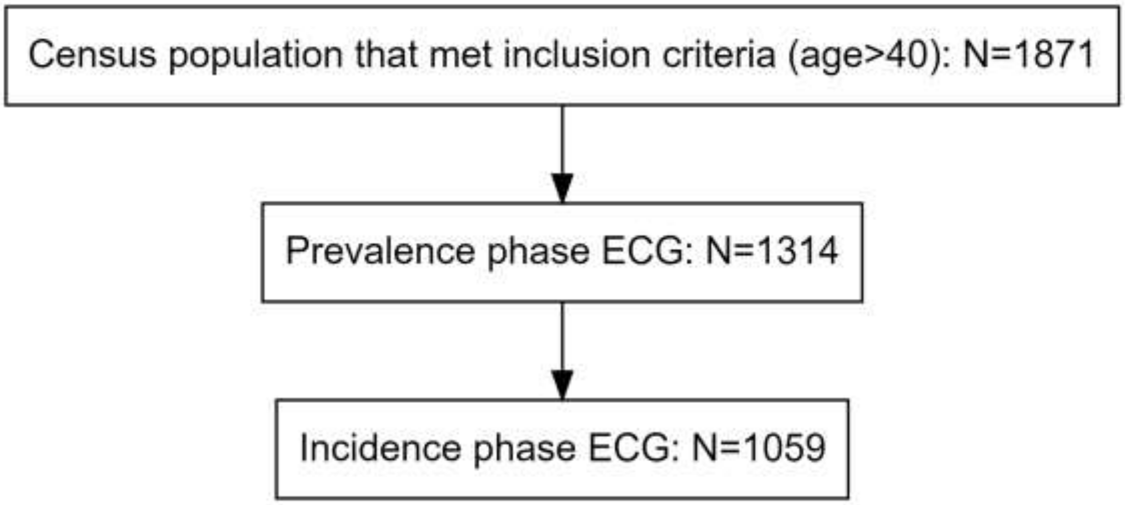

Supplement: Supplementary Figure S1. — STROBE Diagram for Tsimane subject recruitment. N = 1871 refers to all Tsimane who were at least age 40 years from 2005–2019, considering that the THLHP medical team sampled adults age 40+ throughout the entire study period (i.e. not only at baseline). N = 1314 adults had at least one ECG. 557 adults were not sampled, either because ECGs were not measured on all medical rounds (most common, largely due to damaged equipment, lack of electricity, and in some cases lack of time), or because adults were either not present in the village during visits when ECGs assessed (common reason), or were present in the community but did not show up for their clinical visit (less common). 255 adults were lost to follow-up, due either to not being in the village during follow-up visits (most common), being present but not showing up for their clinical visit (less common), or being seen by project physicians but refusing an ECG (rare). [file agh-87-1-3252-s1.pdf]
