# Supplementary material for: Very Low Prevalence and Incidence of Atrial Fibrillation among Bolivian Forager-Farmers
Source: Ann Glob Health. 2021 Feb 16;87(1):18. doi: 10.5334/aogh.3252 (PMC7894370; doi:10.5334/aogh.3252)

Supplementary Figure S2. STROBE Diagram for Moseten subject recruitment. The prevalence phase is based on one medical round in 2015-2016; incidence phase is based on a follow-up visit in 2017-2018. Of the 561 eligible Moseten aged 40+ years, 20 were either not present in the community during THLHP visits (most common), or were present but did not visit our mobile clinic (less common). Loss to follow-up during the second visit was due to not revisiting all of the same villages seen during the prevalence phase, and slightly poorer attendance at our clinic.

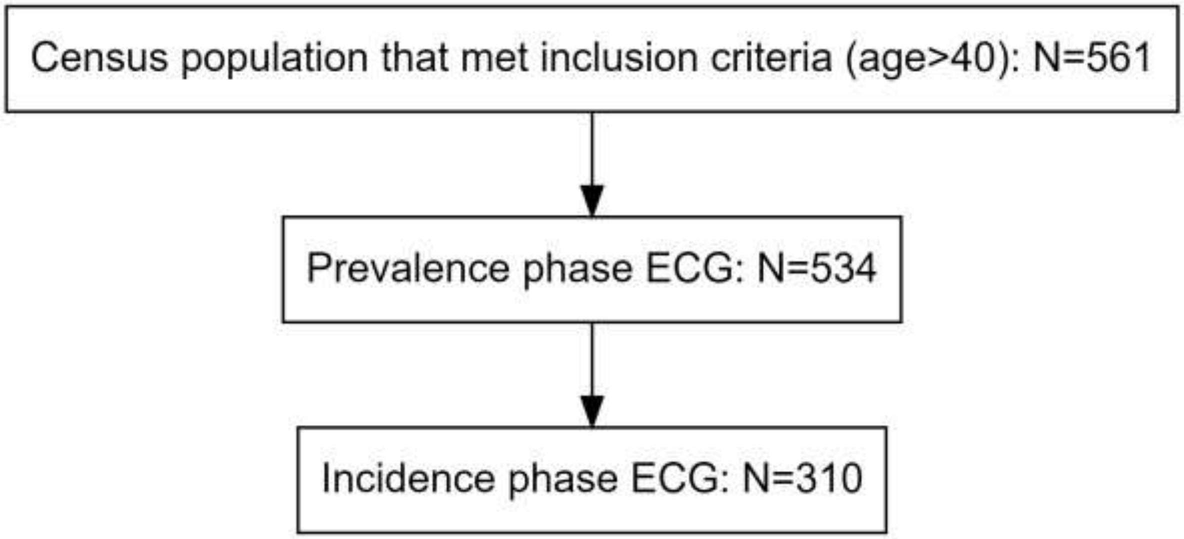

Supplement: Supplementary Figure S2. — STROBE Diagram for Moseten subject recruitment. The prevalence phase is based on one medical round in 2015–2016; incidence phase is based on a follow-up visit in 2017–2018. Of the 561 eligible Moseten aged 40+ years, 20 were either not present in the community during THLHP visits (most common), or were present but did not visit our mobile clinic (less common). Loss to follow-up during the second visit was due to not revisiting all of the same villages seen during the prevalence phase, and slightly poorer attendance at our clinic. [file agh-87-1-3252-s2.pdf]
