# Supplementary material for: Very Low Prevalence and Incidence of Atrial Fibrillation among Bolivian Forager-Farmers
Source: Ann Glob Health. 2021 Feb 16;87(1):18. doi: 10.5334/aogh.3252 (PMC7894370; doi:10.5334/aogh.3252)
Supplement: Supplementary Table S1. — Potential sample bias in prevalence and incidence phases. Biomarker comparisons between those who received or did not receive an ECG during the prevalence phase, and those who did or did not receive a follow-up ECG in the incident phase. Mean values are compared using student t-tests. Blue color indicates lower AF risk among unsampled adults, whereas red color indicates higher AF risk. In the prevalence phase, unsampled Tsimane and Moseten are similar or show lower AF risk than those sampled with an ECG. In the incidence phase, Tsimane from the prevalence phase who were lost to follow-up ECG showed only slightly higher AF risk than those sampled (2.1 years older, 2.6 mg/dL lower HDL, 1.8 mmHg higher systolic blood pressure, 6.5 mm/hr higher ESR), though also show lower AF risk for LDL (14 mg/dL lower) and total cholesterol (13.3 mg/dL lower). Among Moseten, those lost to follow-up showed only slightly higher BMI (0.9 kg/m2 higher). [file agh-87-1-3252-s3.pdf]

Supplementary Table S1. Potential sample bias in prevalence and incidence phases. Biomarker comparisons between those who received or did not receive an ECG during the prevalence phase, and those who did or did not receive a follow-up ECG in the incident phase. Mean values are compared using student t-tests. Blue color indicates lower AF risk among unsampled adults, whereas red color indicates higher AF risk. In the prevalence phase, unsampled Tsimane and Moseten are similar or show lower AF risk than those sampled with an ECG. In the incidence phase, Tsimane from the prevalence phase who were lost to follow-up ECG showed only slightly higher AF risk than those sampled (2.1 years older, 2.6 mg/dL lower HDL, 1.8 mmHg higher systolic blood pressure, 6.5 mm/hr higher ESR), though also show lower AF risk for LDL (14 mg/dL lower) and total cholesterol (13.3 mg/dL lower). Among Moseten, those lost to follow-up showed only slightly higher BMI (0.9 kg/m^2 higher).

	**Prevalence Phase**					**Incidence Phase**				
	No ECG		ECG			No ECG		ECG		
Variable	N	Mean	N	Mean	p-value	N	Mean	N	Mean	p-value
Population: **Tsimane**										
Age	557	49.3	1314	52.7	<0.001	255	54.2	1059	52.3	0.024
LDL	201	83.5	1032	86.7	0.104	108	74.2	924	88.2	<0.001
HDL	206	37.1	1035	37.5	0.456	109	35.1	926	37.7	0.003
Cholesterol	225	141.3	1112	143.3	0.348	130	131.5	982	144.8	<0.001
BMI	494	24.2	1306	24	0.350	250	24.1	1056	24	0.669
Systolic BP	492	111.4	1305	113.9	<0.001	249	115.4	1056	113.6	0.071
Log IL6	90	0.4	740	0.5	0.659	40	0.4	700	0.5	0.389
ESR	423	28.4	1304	31.3	0.002	246	36.6	1058	30.1	<0.001
Population: **Moseten**										
Age	27	45.7	534	55.1	<0.001	224	52.2	310	57.1	<0.001
LDL	3	113.9	363	105.6	0.894	102	110.6	261	103.6	0.130
HDL	3	55.4	363	37.6	0.036	101	36.5	262	38.1	0.120
Cholesterol	3	169.1	368	159.4	0.847	105	163	263	157.9	0.230
BMI	18	26.3	523	25.9	0.658	217	26.4	306	25.5	0.030
Systolic BP	20	118.5	526	120.9	0.366	218	119.9	308	121.6	0.252
Log IL6	3	1.2	114	0.7	0.502	28	0.5	86	0.8	0.117
ESR	3	25.3	449	34.4	0.448	145	31.2	304	35.9	0.026
